# Information Geometry for Covariance Estimation in Heterogeneous Clutter with Total Bregman Divergence

**DOI:** 10.3390/e20040258

**Published:** 2018-04-08

**Authors:** Xiaoqiang Hua, Yongqiang Cheng, Hongqiang Wang, Yuliang Qin

**Affiliations:** School of Electronic Science, National University of Defence Technology, Changsha 410073, China

**Keywords:** covariance matrix estimation, total Bregman divergence, information geometry, adaptive normalized matched filter

## Abstract

This paper presents a covariance matrix estimation method based on information geometry in a heterogeneous clutter. In particular, the problem of covariance estimation is reformulated as the computation of geometric median for covariance matrices estimated by the secondary data set. A new class of total Bregman divergence is presented on the Riemanian manifold of Hermitian positive-definite (HPD) matrix, which is the foundation of information geometry. On the basis of this divergence, total Bregman divergence medians are derived instead of the sample covariance matrix (SCM) of the secondary data. Unlike the SCM, resorting to the knowledge of statistical characteristics of the sample data, the geometric structure of matrix space is considered in our proposed estimators, and then the performance can be improved in a heterogeneous clutter. At the analysis stage, numerical results are given to validate the detection performance of an adaptive normalized matched filter with our estimator compared with existing alternatives.

## 1. Introduction

The estimation of the disturbance covariance matrix is an important subject in the field of advanced radar signal processing. Many algorithms manipulate the covariance matrix of sample data, such as array signal processing [1,2], multichannel signal processing [3,4], adaptive radar detection [5,6,7], and space-time adaptive processing [8,9,10]. The commonly used estimator is the sample covariance matrix (SCM), which is often derived from the maximum-likelihood (ML) of *K N*-dimensional secondary data. A condition is assumed that these *K* secondary data are independent and identical distributed zero-mean complex circular Gaussian vectors. Usually, the solution of this ML exists when the amount of secondary data *K* is greater than the matrix dimension *N* (K≥N). In particular, it can achieve a good performance for K≥2N [11]. Unfortunately, in real heterogeneous clutter, the statistical distribution of the whole environment is very difficult to obtain, since the secondary data could be contaminated by power variations, clutter discretes, and/or outliers. Therefore, it is very necessary and meaningful to obtain the estimation of disturbance matrix that is not relying on the statistical characterization of the whole environment.

Many covariance estimation algorithms derived from the geometry of matrix space, not resorting to the statistical characterization of sample data, are reported in the literature. For instance, the Riemannian mean and median of covariance matrices is proposed to design the radar target detector [12,13,14,15,16]. In [17,18,19,20], the median is used for covariance estimation in many radar processing applications. The Riemannian mean is utilized to estimate the covariance matrix in space-time adaptive processing [21,22,23]. The results have shown that the projection algorithm with Riemannian mean can yield significant performance gains. In [24,25], some geometric barycenter and medians are proposed for radar training data selection in a homogeneous environment. In [26], a geometric method is presented for covariance estimation, where each estimator is associated with a given unitary invariant norm and performs the sample covariance matrix projection into a specific set of structured covariance matrices. Recently, geometric Barycenters of symmetric positive definite matrices have been used for covariance estimators in compound-Gaussian clutter [27]. Moreover, in our previous work [28,29], we have proposed a lot of divergence means and medians of covariance matrices computed in a neighborhood of the cell under test for radar target detection. Finally, the geometric approach is used also in many other applications; for instance, the Bhattacharyya mean and median are exploited for diffusion tensor magnetic resonance (DT-MRI) image segmentation [30,31]. In these contexts, the geometric approaches have achieved good performances.

In this paper, a new class of total Bregman divergence (tBD) is presented on the Riemannian manifold. Based on the tBD, the three geometric medians, including total square loss (TSL) median, total von Neumann (TVN) median, and total LogDet (TLD) median, are derived, and are used as the estimators of disturbance matrix. As a matter of fact, the SCM of *K* secondary data can be seen as the arithmetic mean of *K* autocovariance matrices of rank one. The arithmetic mean ignores the fact that these matrices lie in a nonlinear matrix space, the Riemannian manifold, which is the foundation of information geometry. As the geometric medians are not relying on the statistical characteristics of the sample data in heterogeneous clutter, the performance of covariance estimation can be improved.

The rest of this paper is organized as follows: Section 2 formulates the problem of covariance estimation from information geometry; the total Bregman divergence-based estimators are presented in Section 3; Section 4 gives the theoretical analysis about the robustness of the proposed estimators; experimental results are presented in Section 5; Section 6 concludes our work.

**Notation**: Here are some notations for the description of this article. A matrix A and a vector x are noted as uppercase bold and lowercase bold, respectively. The conjugate transpose of matrix A is denoted as $A^{†}$. tr(A) is the trace of matrix A. det(A) is the determinant of matrix A. I denotes the identity matrix. Finally, E(·) denotes statistical expectation.

## 2. Problem Formulated from Information Geometry

In this section, a heterogeneous environment is considered for covariance estimation. The secondary data $r_{k},k=1,2,\ldots ,K$ is a spherically invariant random vector, and can be expressed as,

(1) $$\begin{array}{c} r_{k}=\sqrt{\tau_{k}}g_{k},k=1,\ldots ,K, \end{array}$$

where $\tau_{k}$ is a nonnegative scalar random variable, and $g_{k}$ is a *N*-dimensional circularly symmetric zero-mean vectors with an arbitrary joint statistical distribution and sharing the same covariance matrix,

(2) $$\begin{array}{c} E[g_{k}g_{k}^{†}]=\Sigma ,k=1,\ldots ,K, \end{array}$$

where † denotes the conjugate transpose. Since the knowledge of the statistical characterization of the secondary data is not known in heterogeneous clutter, the classic approaches, e.g., ML and minimum mean-square error, cannot be applied for covariance estimation of the sample data. Thus, other covariance estimators, not dependent on the probability distribution of the whole environment, are very promising. In this paper, a covariance estimation method based on information geometry is proposed.

Recall that the SCM of *K* secondary data $r_{k},k=1,2,\ldots ,K$ can be given as

(3) $$\begin{array}{c} \hat{\Sigma }=\frac{1}{K}\sum_{k=1}^{K}r_{k}r_{k}^{†}, \end{array}$$

where $r_{k}r_{k}^{†}$ is an autocovariance matrix of rank one, and is singular. It can be noted from Equation (3) that $\hat{\Sigma }$ is the arithmetic mean of *K* autocovariance matrices. In fact, these matrices lie in the nonlinear Hermitian matrix space, as the sample data is complex. It is well known that Hermitian positive-definite (HPD) matrices form a differentiable Riemannian (and also a Finsler) manifold [32,33], that is the most studied example of a manifold with nonpositive curvature [34,35]. In order to facilitate the analysis, the singular matrix $r_{k}r_{k}^{†}$ is positive by adding an identity matrix, as $R_{k}$ = $r_{k}r_{k}^{†}$ + I. Then, the disturbance covariance matrix can be estimated by a median related to a divergence on the Riemannian manifold of HPD matrices.

**Figure 1:**
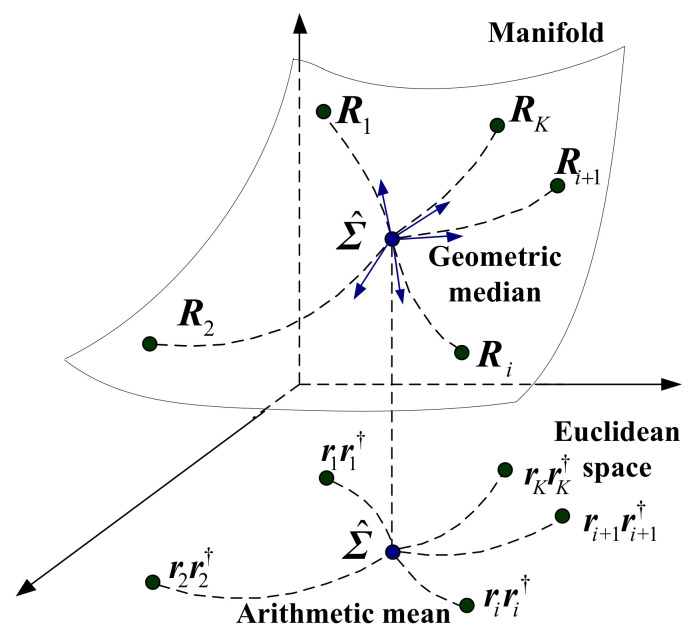
The arithmetic mean and geometric median.

As illustrated in Figure 1, the geometric median is performed on the Riemannian manifold of HPD matrices with a non-Euclidean metric, whereas the arithmetic mean is considered in the Euclidean space. The difference implies that the different geometric structures are considered in these two estimators. Moreover, for different metrics on the Riemannian manifold, the performance of covariance estimation may be very different. These results will be found in our other reports [36]. In the next section, a new class of tBD is proposed on the Riemannian manifold. On basis of the tBD, the tBD median is derived and used for the estimator.

## 3. Total Bregman Divergence-Based Estimators on the Manifold

In this section, a new class of tBD is proposed on the Riemannian manifold. Then, the medians associated with the tBD are derived.

### 3.1. The Geometry of HPD Matrices

Let $H\left(n\right)=\left\{A|A=A^{H}\right\}$ denote the space of n×n Hermitian matrix. For a Hermitian matrix A, if the quadratic form $x^{H}Ax>0,\forall x\in C\left(n\right)$, then A is an HPD matrix, where C(n) is the space of *n*-dimensional complex vectors. All n×n HPD matrices consist of a positive-definite Hermitian matrix space P(n),

(4) $$\begin{array}{c} P(n)=\{A|A>0,A\in H(n)\}, \end{array}$$

which forms a Riemannian manifold of dimension n(n+1)/2 with a constant non-positive curvature [35]. For a point A on the Riemannian manifold, the infinitesimal arclength between A and A+dA is given by

(5) $$\begin{array}{c} ds:={\left(tr{\left(A^{-1}dA\right)}^{2}\right)}^{1/2}={∥A^{-1/2}dAA^{-1/2}∥}_{F}, \end{array}$$

where ds defines a metric on the Riemannian manifold [37]. ${∥.∥}_{F}$ is the Frobenius norm of a matrix. The inner product and corresponding norm on the tangent space at the point A can be defined as [38]

(6) $$\begin{array}{c} {\langle P_{1},P_{2}\rangle }_{A}=tr\left(A^{-1}P_{1}A^{-1}P_{2}\right),{∥P_{1}∥}_{A}={\langle P_{1},P_{2}\rangle }_{A}^{1/2}. \end{array}$$

For two points $P_{1}$ and $P_{2}$ on the Riemannian manifold, the affine invariant (Riemannian) distance is given by [39]

(7) $$\begin{array}{c} d_{R}^{2}\left(P_{1},P_{2}\right)={∥logm\left(P_{1}^{-1/2}P_{2}P_{1}^{-1/2}\right)∥}_{F}^{2}, \end{array}$$

where logm is the logarithmic map on the Riemannian manifold of HPD matrices.

### 3.2. Total Bregman Divergence

The tBD has been proposed on the space of convex functions by Baba C. Vemuri, and has been used for DT-MRI analysis [40], shape retrieval [41,42], and object tracking [43]. For x,y∈R, *f* is a differentiable, strictly convex function, the total Bregman divergence δ between *x*, *y* is defined as [40]

(8) $$\begin{array}{c} \delta_{f}\left(x,y\right)=\frac{f\left(x\right)-f\left(y\right)-f^{\prime }\left(y\right)\left(x-y\right)}{\sqrt{1+{∥f^{\prime }\left(y\right)∥}^{2}}}, \end{array}$$

where $f^{\prime }\left(y\right)$ is the derivative of f(x) at *y*.

**Figure 2:**
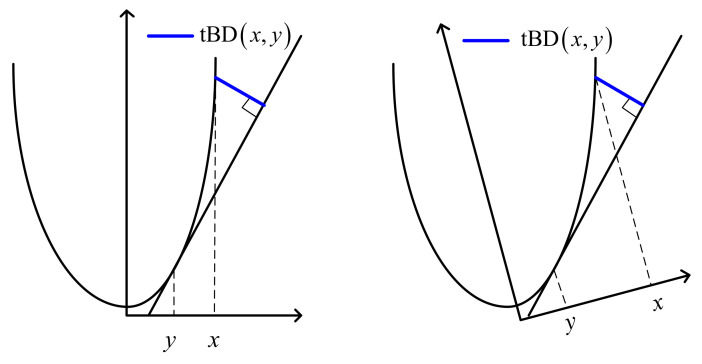
The geometric definition of total Bregman divergence.

As illustrated in Figure 2, the tBD between *x* and *y* is defined as the orthogonal distance between the value of a convex and differentiable function *f* at the first argument of *y* and its tangent at the second argument. It can be also noted from Figure 2 that when the coordinate system rotates at an angle, the tBD between *x* and *y* does not change. This implies that the tBD is invariant to linear transformation; in other words, it is statistically robust.

In the following, we extend the definition of tBD to the Riemannian manifold of HPD matrices.

**Definition** **1.** *Let f be a strictly convex and differentiable function, for two HPD matrices X, Y, the tBD is defined as*

(9) $$\begin{array}{c} \delta_{f}\left(X,Y\right)=\frac{tr\left(f\left(X\right)\right)-tr\left(f\left(Y\right)\right)-tr\left(f^{\prime }\left(Y\right)\left(X-Y\right)\right)}{\sqrt{1+{∥f^{\prime }\left(Y\right)∥}_{F}^{2}}}. \end{array}$$

In the following, we give the definitions of the three new divergences when the function *f* has various forms.

Let $f\left(x\right)=x^{2}$, then $tr\left(f\left(X\right)\right)=X^{2}$, $f^{\prime }\left(Y\right)=2Y$, and Equation (9) denotes the TSL

(10) $$\begin{array}{c} \delta_{f}\left(X,Y\right)=\frac{tr\left(X^{2}-Y^{2}-2Y\left(X-Y\right)\right)}{\sqrt{1+{∥2Y∥}_{F}^{2}}}. \end{array}$$

If $f\left(x\right)=x\log x-x$, then $tr\left(f\left(X\right)\right)=tr\left(X\log X-X\right)$, $f^{\prime }\left(Y\right)=\log Y$, and Equation (9) yields the divergence, which is called the TVN,

(11) $$\begin{array}{c} \delta_{f}\left(X,Y\right)=\frac{tr\left(X\log X-X\log Y-X+Y\right)}{\sqrt{1+{∥\log Y∥}_{F}^{2}}}. \end{array}$$

When $f\left(x\right)=-\log x$, then $tr\left(f\left(X\right)\right)=-\log\det\left(X\right)$, $f^{\prime }\left(Y\right)=Y^{-1}$, and we obtain the divergence referred to as the TLD or the total Stein loss,

(12) $$\begin{array}{c} \delta_{f}\left(X,Y\right)=\frac{tr\left(Y^{-1}\left(X-Y\right)-\log X+\log Y\right)}{\sqrt{1+{∥Y^{-1}∥}_{F}^{2}}}. \end{array}$$

Based on the three divergences on the Riemannian manifold, the medians for a finite set of HPD matrices are derived in the next section.

### 3.3. Total Bregman Divergence Median for HPD Matrices

For a set of HPD matrices, the median related to a measure is defined as the minimizer of the sum of the distance to the given matrices.

**Definition** **2.** *Let f be a differentiable convex function; then, the median related to the tBD Equation (9), of m HPD matrices $\left\{R_{1},R_{2},\ldots ,R_{m}\right\}$ is defined as*

(13) $$\begin{array}{c} \hat{R}=\underset{R}{\arg\min}\;\sum_{i=1}^{m}\delta_{f}\left(R,R_{i}\right). \end{array}$$

It is worth pointing out that $\hat{R}$ will be the arithmetic mean, if the Frobenius norm, instead of the tBD, is utilized.

**Proposition** **1.** The median for a finite set of HPD matrices with respect to the tBD exists, and is unique.

**Proof of Proposition** **1.** *To find the median $\hat{R}$, let $F\left(R\right)=\sum_{i=1}^{m}\delta_{f}\left(R,R_{i}\right)$ denote the objection function*

(14) $$\begin{array}{c} F\left(R\right)=\sum_{i=1}^{m}\frac{tr\left(f\left(R\right)\right)-tr\left(f\left(R_{i}\right)\right)-tr\left(f^{\prime }\left(R_{i}\right)\left(R-R_{i}\right)\right)}{\sqrt{1+{∥f^{\prime }\left(R_{i}\right)∥}_{F}^{2}}}. \end{array}$$

Setting the gradient $\nabla F\left(R\right)$ equal to zero,

(15) $$\begin{array}{c} \nabla F\left(R\right)=\sum_{i=1}^{m}\frac{f^{\prime }\left(R\right)-f^{\prime }\left(R_{i}\right)}{\sqrt{1+{∥f^{\prime }\left(R_{i}\right)∥}_{F}^{2}}}=0. \end{array}$$

Solving Equation (15) yields

(16) $$\begin{array}{c} f^{\prime }\left(\hat{R}\right)=\sum_{i=1}^{m}\frac{f^{\prime }\left(R_{i}\right)}{\sqrt{1+{∥f^{\prime }\left(R_{i}\right)∥}_{F}^{2}}}/\sum_{i=1}^{m}\frac{1}{\sqrt{1+{∥f^{\prime }\left(R_{i}\right)∥}_{F}^{2}}}. \end{array}$$

The tBD $\delta_{f}\left(R,R_{i}\right)$ is a convex function, and $F\left(R\right)$, the sum of *m* convex functions, is also a convex function. Hence, the median $\hat{R}$ indeed exists. Moreover, since *f* is a convex function, and $f^{\prime }$ is monotonic, the median $\hat{R}$ is unique.

In the following, three concrete cases of the tBD are derived when *f* is given as different forms.

**Proposition** **2.** *The median related to the TSL (10), of m HPD matrices $\left\{R_{1},R_{2},\ldots ,R_{m}\right\}$ is given by*

(17) $$\begin{array}{c} \hat{R}=\sum_{i=1}^{m}\mu_{i}R_{i},\mu_{i}=\frac{w_{i}}{\sum_{i=1}^{m}w_{i}},w_{i}=\frac{1}{\sqrt{1+{∥2R_{i}∥}_{F}^{2}}}. \end{array}$$

**Proposition** **3.** *The median related to the TVN divergence (11) of m HPD matrices $\left\{R_{1},R_{2},\ldots ,R_{m}\right\}$ is given by*

(18) $$\begin{array}{c} \hat{R}=\exp\left(\sum_{i=1}^{m}\mu_{i}\log\left(R_{i}\right)\right),\mu_{i}=\frac{w_{i}}{\sum_{i=1}^{m}w_{i}},w_{i}=\frac{1}{\sqrt{1+{∥\log\left(R_{i}\right)∥}_{F}^{2}}}. \end{array}$$

**Proposition** **4.** *The median related to the TLD divergence (12), of m HPD matrices $\left\{R_{1},R_{2},\ldots ,R_{m}\right\}$ is given by*

(19) $$\begin{array}{c} \hat{R}={\left(\sum_{i=1}^{m}\mu_{i}R_{i}^{-1}\right)}^{-1},\mu_{i}=\frac{w_{i}}{\sum_{i=1}^{m}w_{i}},w_{i}=\frac{1}{\sqrt{1+{∥R_{i}^{-1}∥}_{F}^{2}}}. \end{array}$$

**Proof of Proposition 2, 3, and** **4.** *With the help of the formulas $\partial \left(R^{2}\right)/\partial \;R=2R$, $\partial \left(R\log R-R\right)/\partial \;R=\log R$, and $\partial \left(-\mathrm{logdet}\left(R\right)\right)/\partial \;R=-R^{-1}$, we then have*

(20) $$\begin{array}{c} \nabla F\left(R\right)=\sum_{i=1}^{m}\frac{2R-2R_{i}}{\sqrt{1+{∥2R_{i}∥}_{F}^{2}}}, \end{array}$$

(21) $$\begin{array}{c} \nabla F\left(R\right)=\sum_{i=1}^{m}\frac{\log\left(R\right)-\log\left(R_{i}\right)}{\sqrt{1+{∥\log\left(R_{i}\right)∥}_{F}^{2}}}, \end{array}$$

(22) $$\begin{array}{c} \nabla F\left(R\right)=\sum_{i=1}^{m}\frac{R^{-1}-R_{i}^{-1}}{\sqrt{1+{∥R_{i}^{-1}∥}_{F}^{2}}}. \end{array}$$

These three equations are for the three propositions above in the same order. Equating these gradients to zero and solving for R yield the medians.

## 4. Robustness Analysis of Total Bregman Divergence Median

This section is devoted to analyzing the robustness of total Bregman divergence median and arithmetic mean via the influence function. Let $\bar{R}$ be the median, associated with total Bregman divergence, of *m* HPD matrices $\left\{R_{1},\ldots ,R_{m}\right\}$. $\tilde{R}$ is the median by adding a set of *n* outliers $\left\{Q_{1},\ldots ,Q_{n}\right\}$ with a weight ε(ε≪1) to $\left\{R_{1},\ldots ,R_{m}\right\}$. Then, we can define $\tilde{R}=\bar{R}+\varepsilon H\left(Q\right)$, and H(Q) denotes the influence function. In the following, four propositions are presented.

**Proposition** **5.** *The influence function of arithmetic mean related to the Frobenius norm, of m HPD matrices $\left\{R_{1},\ldots ,R_{m}\right\}$ and n outliers $\left\{Q_{1},\ldots ,Q_{n}\right\}$ is given by*

(23) $$\begin{array}{c} H\left(Q\right)=\frac{1}{n}\sum_{i=1}^{n}Q_{i}-\bar{R}. \end{array}$$

**Proof of Proposition** **5.** *Let F(R) be the objection function*

(24) $$\begin{array}{c} F\left(R\right)=\left(1-\varepsilon \right)\frac{1}{m}\sum_{i=1}^{m}∥R-R_{i}{∥}_{F}^{2}+\varepsilon \frac{1}{n}\sum_{j=1}^{n}∥R-Q_{j}{∥}_{F}^{2}. \end{array}$$

The derivative of objection function F(R) is

(25) $$\begin{array}{c} \nabla F\left(R\right)=\left(1-\varepsilon \right)\frac{1}{m}\sum_{i=1}^{m}2\left(R-R_{i}\right)+\varepsilon \frac{1}{n}\sum_{j=1}^{n}2\left(R-Q_{j}\right). \end{array}$$

Note that $\tilde{R}$ is the median of *m* matrices and *n* outliers, and $\bar{R}$ is the median of *m* matrices; then, we have

(26) $$\begin{array}{cc} & \tilde{R}=\underset{R}{\arg\min}\;F\left(R\right)\\ \Rightarrow & \nabla F\left(\tilde{R}\right)=\left(1-\varepsilon \right)\frac{1}{m}\sum_{i=1}^{m}2\left(\tilde{R}-R_{i}\right)+\varepsilon \frac{1}{n}\sum_{j=1}^{n}2\left(\tilde{R}-Q_{j}\right)=0 \end{array}$$

and

(27) $$\begin{array}{cc} & \bar{R}=\underset{R}{\arg\min}\;G\left(R\right),G\left(R\right)=\frac{1}{m}\sum_{i=1}^{m}{∥R-R_{i}∥}_{F}^{2}\\ \Rightarrow & \nabla F\left(\bar{R}\right)=\frac{1}{m}\sum_{i=1}^{m}2\left(\bar{R}-R_{i}\right)=0. \end{array}$$

Substitute $\tilde{R}=\bar{R}+\varepsilon H\left(Q\right)$ into Equation (26), and we have

(28) $$\begin{array}{cc} & 2\left(1-\varepsilon \right)\frac{1}{m}\sum_{i=1}^{m}\left(\bar{R}+\varepsilon H\left(Q\right)-R_{i}\right)+2\varepsilon \frac{1}{n}\sum_{j=1}^{n}\left(\bar{R}+\varepsilon H\left(Q\right)-Q_{j}\right)=0\\ \Rightarrow & \left(1-\varepsilon \right)\frac{1}{m}\sum_{i=1}^{m}\left(\bar{R}-R_{i}\right)+\left(1-\varepsilon \right)\varepsilon H\left(Q\right)+\varepsilon \frac{1}{n}\sum_{j=1}^{n}\left(\bar{R}+\varepsilon H\left(Q\right)-Q_{j}\right)=0\\ \Rightarrow & \varepsilon H\left(Q\right)-\varepsilon^{2}H\left(Q\right)+\varepsilon \frac{1}{n}\sum_{j=1}^{n}\left(\bar{R}-Q_{j}\right)+\varepsilon^{2}H\left(Q\right)=0\\ \Rightarrow & H\left(Q\right)=\frac{1}{n}\sum_{j=1}^{n}\left(Q_{j}-\bar{R}\right). \end{array}$$

**Proposition** **6.** *The influence function of TSL median related to TSL, of m HPD matrices $\left\{R_{1},\ldots ,R_{m}\right\}$ and n outliers $\left\{Q_{1},\ldots ,Q_{n}\right\}$ is given by*

(29) $$\begin{array}{c} H\left(Q\right)=\frac{m}{n}\sum_{j=1}^{n}\frac{\left(Q_{j}-\bar{R}\right)}{\sqrt{1+{∥2Q_{j}∥}_{F}^{2}}}{\left(\sum_{i=1}^{m}\frac{1}{\sqrt{1+{∥2R_{i}∥}_{F}^{2}}}\right)}^{-1}. \end{array}$$

**Proposition** **7.** *The influence function of TVN median related to TVN, of m HPD matrices $\left\{R_{1},\ldots ,R_{m}\right\}$ and n outliers $\left\{Q_{1},\ldots ,Q_{n}\right\}$ is given by*

(30) $$\begin{array}{c} H\left(Q\right)=\frac{m}{n}\sum_{j=1}^{n}\frac{\left(\log\left(Q_{j}\right)-\log\left(\bar{R}\right)\right)\bar{R}}{\sqrt{1+{∥\log\left(Q_{j}\right)∥}_{F}^{2}}}{\left(\sum_{i=1}^{m}\frac{1}{\sqrt{1+{∥\log\left(R_{i}\right)∥}_{F}^{2}}}\right)}^{-1}. \end{array}$$

**Proposition** **8.** *The influence function of TLD median related to TLD, of m HPD matrices $\left\{R_{1},\ldots ,R_{m}\right\}$ and n outliers $\left\{Q_{1},\ldots ,Q_{n}\right\}$ is given by*

(31) $$\begin{array}{c} H\left(Q\right)=\frac{m}{n}\sum_{j=1}^{n}\frac{\left({\bar{R}}^{-1}-Q_{j}^{-1}\right){\bar{R}}^{2}}{\sqrt{1+{∥Q_{j}^{-1}∥}_{F}^{2}}}{\left(\sum_{i=1}^{m}\frac{1}{\sqrt{1+{∥R_{i}^{-1}∥}_{F}^{2}}}\right)}^{-1}. \end{array}$$

**Proof of Proposition 6, 7, and** **8.** *According to Equation (9), let F(R) be the objection function*

(32) $$\begin{array}{c} F\left(R\right)=\left(1-\varepsilon \right)\frac{1}{m}\sum_{i=1}^{m}\delta_{f}\left(R,R_{i}\right)+\varepsilon \frac{1}{n}\sum_{j=1}^{n}\delta_{f}\left(R,Q_{j}\right). \end{array}$$

Note that $\tilde{R}$ is the median of *m* matrices and *n* outliers; then, we have,

(33) $$\begin{array}{cc} & \nabla F(\tilde{R})=0\\ \Rightarrow & \left(1-\varepsilon \right)\frac{1}{m}\sum_{i=1}^{m}\frac{f^{\prime }\left(\tilde{R}\right)-f^{\prime }\left(R_{i}\right)}{\sqrt{1+{∥f^{\prime }\left(R_{i}\right)∥}_{F}^{2}}}+\varepsilon \frac{1}{n}\sum_{j=1}^{n}\frac{f^{\prime }\left(\tilde{R}\right)-f^{\prime }\left(Q_{j}\right)}{\sqrt{1+{∥f^{\prime }\left(Q_{j}\right)∥}_{F}^{2}}}=0. \end{array}$$

Using Taylor expansions on $\tilde{R}=\bar{R}+\varepsilon H\left(Q\right)$ on the derivative function $f^{\prime }$, we can obtain

(34) $$\begin{array}{c} f^{\prime }\left(\tilde{R}\right)=f^{\prime }\left(\bar{R}\right)+\left(\tilde{R}-\bar{R}\right)f^{\prime \prime }\left(\bar{R}\right)=f^{\prime }\left(\bar{R}\right)+\varepsilon H\left(Q\right)f^{\prime \prime }\left(\bar{R}\right). \end{array}$$

Substituting Equation (34) into Equation (33),

(35) $$\begin{array}{c} \left(1-\varepsilon \right)\frac{1}{m}\sum_{i=1}^{m}\frac{f^{\prime }\left(\bar{R}\right)+\varepsilon H\left(Q\right)f^{\prime \prime }\left(\bar{R}\right)-f^{\prime }\left(R_{i}\right)}{\sqrt{1+{∥f^{\prime }\left(R_{i}\right)∥}_{F}^{2}}}+\varepsilon \frac{1}{n}\sum_{j=1}^{n}\frac{f^{\prime }\left(\bar{R}\right)+\varepsilon H\left(Q\right)f^{\prime \prime }\left(\bar{R}\right)-f^{\prime }\left(Q_{j}\right)}{\sqrt{1+{∥f^{\prime }\left(Q_{j}\right)∥}_{F}^{2}}}=0. \end{array}$$

As $\bar{R}$ is the median of *m* matrices, we have

(36) $$\begin{array}{c} \frac{1}{m}\sum_{i=1}^{m}\frac{f^{\prime }\left(\bar{R}\right)-f^{\prime }\left(R_{i}\right)}{\sqrt{1+{∥f^{\prime }\left(R_{i}\right)∥}_{F}^{2}}}=0. \end{array}$$

Substituting Equation (36) into Equation (35), and ignoring the term containing $\varepsilon^{2}$,

(37) $$\begin{array}{c} \frac{1}{m}\sum_{i=1}^{m}\frac{\varepsilon H\left(Q\right)f^{\prime \prime }\left(\bar{R}\right)}{\sqrt{1+{∥f^{\prime }\left(R_{i}\right)∥}_{F}^{2}}}+\varepsilon \frac{1}{n}\sum_{j=1}^{n}\frac{f^{\prime }\left(\bar{R}\right)-f^{\prime }\left(Q_{j}\right)}{\sqrt{1+{∥f^{\prime }\left(Q_{j}\right)∥}_{F}^{2}}}=0. \end{array}$$

Finally, the influence function of total Bregman divergence median can be derived

(38) $$\begin{array}{c} H\left(Q\right)=-\frac{m}{n}\sum_{j=1}^{n}\frac{f^{\prime }\left(\bar{R}\right)-f^{\prime }\left(Q_{j}\right)}{\sqrt{1+{∥f^{\prime }\left(Q_{j}\right)∥}_{F}^{2}}}{\left(\sum_{i=1}^{m}\frac{f^{\prime \prime }\left(\bar{R}\right)}{\sqrt{1+{∥f^{\prime }\left(R_{i}\right)∥}_{F}^{2}}}\right)}^{-1}. \end{array}$$

Let $f\left(R\right)=R^{2}$; then, $f^{\prime }\left(R\right)=2R$ and $f^{\prime \prime }\left(R\right)=2$. Substitute these into Equation (38), and we can obtain **Proposition** 6.

When $f\left(R\right)=R\log\left(R\right)-R$, then $f^{\prime }\left(R\right)=\log\left(R\right)$ and $f^{\prime \prime }\left(R\right)=R^{-1}$. Substitute these into Equation (38), and we can obtain **Proposition** 7.

If $f\left(R\right)=-\log\left(R\right)$, then $f^{\prime }\left(R\right)=-R^{-1}$ and $f^{\prime \prime }\left(R\right)=R^{-2}$. Substitute these into Equation (38), and we can obtain **Proposition** 8.

In addition, we show that the influence value of total Bregman divergence median has an upper bound when the number of outlier n=1. Let

(39) $$\begin{array}{c} w\left(R_{i}\right)=\sqrt{1+{∥f^{\prime }\left(R_{i}\right)∥}_{F}^{2}},G=\frac{1}{m}\sum_{i=1}^{m}\frac{f^{\prime \prime }\left(\bar{R}\right)}{w\left(R_{i}\right)}. \end{array}$$

Then, Equation (38) can be rewritten as

(40) $$\begin{array}{c} H\left(Q\right)=\frac{f^{\prime }\left(Q\right)-f^{\prime }\left(\bar{R}\right)}{\sqrt{1+{∥f^{\prime }\left(Q\right)∥}_{F}^{2}}}{\left(\frac{1}{m}\sum_{i=1}^{m}\frac{f^{\prime \prime }\left(\bar{R}\right)}{\sqrt{1+{∥f^{\prime }\left(R_{i}\right)∥}_{F}^{2}}}\right)}^{-1}=\frac{f^{\prime }\left(Q\right)-f^{\prime }\left(\bar{R}\right)}{w\left(Q\right)}G^{-1}. \end{array}$$

Note that $w\left(Q\right)=\sqrt{1+{∥f^{\prime }\left(Q\right)∥}_{F}^{2}}\geq {∥f^{\prime }\left(Q\right)∥}_{F}$ and $w\left(Q\right)\geq 1$; then, we have the following inequality:

(41) $$\begin{array}{c} {∥H\left(Q\right)∥}_{F}=\frac{{∥f^{\prime }\left(Q\right)-f^{\prime }\left(\bar{R}\right)∥}_{F}}{w\left(Q\right)}G^{-1}=\frac{{∥f^{\prime }\left(Q\right)-f^{\prime }\left(\bar{R}\right)∥}_{F}}{\sqrt{1+{∥f^{\prime }\left(Q\right)∥}_{F}^{2}}}G^{-1}\\ \;\leq \frac{{∥f^{\prime }\left(Q\right)-f^{\prime }\left(\bar{R}\right)∥}_{F}}{{∥f^{\prime }\left(Q\right)∥}_{F}}G^{-1}\leq \frac{{∥f^{\prime }\left(Q\right)∥}_{F}+{∥f^{\prime }\left(\bar{R}\right)∥}_{F}}{{∥f^{\prime }\left(Q\right)∥}_{F}}G^{-1}\\ \;=\left(1+\frac{{∥f^{\prime }\left(\bar{R}\right)∥}_{F}}{{∥f^{\prime }\left(Q\right)∥}_{F}}\right)G^{-1}\leq \left(1+{∥f^{\prime }\left(\bar{R}\right)∥}_{F}\right)G^{-1}=c, \end{array}$$

where *c* is a constant. From the inequality (41), we can know that the influence function H(Q) has an upper bound when Q varies. It implies that our proposed tBD medians are robust.

## 5. Numerical Simulations

In this section, the performances of adaptive normalized matched filter with the proposed estimators and the normalized sample covariance matrix (NSCM) are evaluated by means of the standard Monte Carlo techniques, as the analytical expression for the probability of detection is not available. Particularly, consider a classical target detection problem, which is formulated as follows:

(42) $$\begin{array}{c} \left\{\begin{array}{c} H_{0}:\left\{\begin{array}{c} z=r\\ z_{k}=r_{k},\;k=1\;\cdots ,K \end{array}\right.\\ H_{1}:\left\{\begin{array}{c} z=\beta s+r\\ z_{k}=r_{k},\;k=1,\;\cdots ,K \end{array}\right. \end{array}\right. \end{array}$$

where z and r denote the received signal and the clutter data in the cell under test, respectively. β is an unknown complex quantity, accounting for the target radar cross section and the channel propagation effects. s is the target steering vector,

(43) $$\begin{array}{c} s=\frac{1}{\sqrt{N}}{\left[1,\exp\left(j2\pi f_{d}\right),\ldots ,\exp\left(j2\pi \left(N-1\right)f_{d}\right)\right]}^{T}, \end{array}$$

where $f_{d}$ is the normalized Doppler frequency. The terms r and $r_{k},$k=1,⋯,K are the spherically invariant random vectors, and can be formulated as,

(44) $$\begin{array}{c} r=\sqrt{\tau }g,\;r_{k}=\sqrt{\tau_{k}}g_{k},\;k=1,\;\cdots ,K, \end{array}$$

where g and $g_{k}$ are *N*-dimensional circularly symmetric zero-mean Gaussian vectors, and share the same covariance matrix Σ. τ and $\tau_{k}$ are positive and real random variables, which are also independent and identical distributed. In particular, the terms τ and $\tau_{k}$ are assumed to follow the inverse gamma distribution,

(45) $$\begin{array}{c} f\left(x\right)=\frac{\beta^{\alpha }}{\Gamma \left(\alpha \right)}x^{-\alpha -1}\exp\left(-\frac{\beta }{x}\right),x\geq 0, \end{array}$$

where α and β denote the shape and scale parameters, respectively. $\Gamma \left(\cdot \right)$ is the gamma function.

For the target detection problem (42), the adaptive normalized matched filtering (ANMF) is often used, which can be given by

(46) $$\begin{array}{c} \frac{s^{†}{\hat{\Sigma }}^{-1}r}{\left(s^{†}{\hat{\Sigma }}^{-1}s\right)\left(r^{†}{\hat{\Sigma }}^{-1}r\right)}\underset{H_{0}}{\overset{H_{1}}{≷}}\gamma , \end{array}$$

where $\hat{\Sigma }$ is an estimator that is based on the secondary data $r_{k},\;k=1\;\cdots ,K$. γ denotes the threshold, which is derived by the Monte Carlo method in order to maintain the false alarm constant. In addition, the covariance matrix of g and $g_{k}$ are given as the sum of two parts,

(47) $$\begin{array}{c} \Sigma =\Sigma_{0}+I, \end{array}$$

where I is accounting for the thermal noise. $\Sigma_{0}$ is related to the clutter, modeled as,

(48) $$\begin{array}{c} \Sigma_{0}\left(i,k\right)=\sigma_{c}^{2}\rho^{\left|i-k\right|}e^{j2\pi f_{dc}\left(i-k\right)},\;i,k=1\;\cdots ,N, \end{array}$$

where ρ is the one-lag correlation coefficient. $\sigma_{c}$ is the clutter-to-noise power ratio. $f_{dc}$ is the clutter normalized Doppler frequency. In this simulation, we set ρ = 0.9, $f_{dc}$ = 0.1, and $\sigma_{c}^{2}$ = 25 dB. The parameters α = 3, and β = 1.

**Figure 3:**
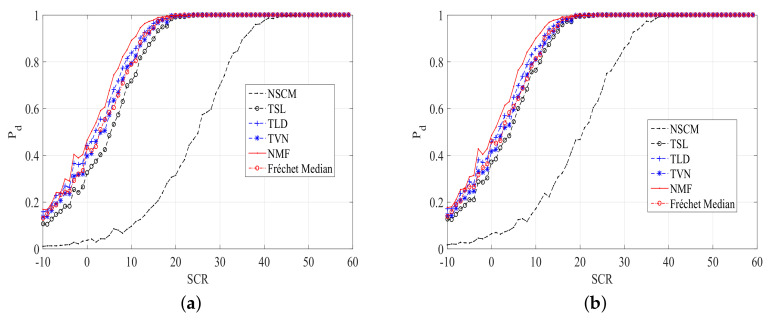
Pd versus SCR plots of ANMFs with the proposed estimators and the NSCM estimator, N=8,K=10. (**a**) $P_{fa}=10^{-5}$; (**b**) $P_{fa}=10^{-4}$.

**Figure 4:**
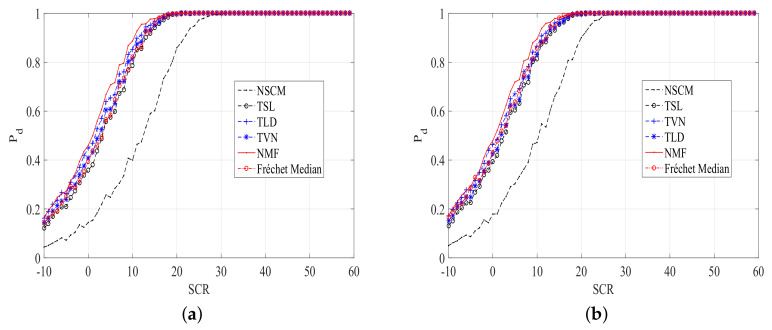
Pd versus SCR plots of ANMFs with the proposed estimators and the NSCM estimator, N=8,K=16. (**a**) $P_{fa}=10^{-5}$; (**b**) $P_{fa}=10^{-4}$.

Performances of ANMFs with our proposed estimators are compared with the Frechet median estimator [18] and the NSCM estimator. The plots of $P_{d}$ versus signal-to-clutter ratio (SCR) for different size of *K*, the number of secondary data, are shown in Figure 3 and Figure 4. The $P_{d}$ is estimated by the relative frequencies of 1000 simulations. The detection threshold is obtained using $100/P_{fa}$ Monte Carlo trials, for a fixed nominal $P_{fa}$. It can be noted from Figure 3 and Figure 4 that the detection performances of ANMFs with our proposed estimators and the Frechet median estimator outperform that of ANMF with the NSCM estimator. The ANMF with the TLD estimator has the best performance, followed by the TVN estimator. The Frechet median estimator has similar performance to the TVN estimator. However, the difference is not very clear. This implies that our proposed estimators are robust for the size of *K*, unlike the NSCM estimator.

**Figure 5:**
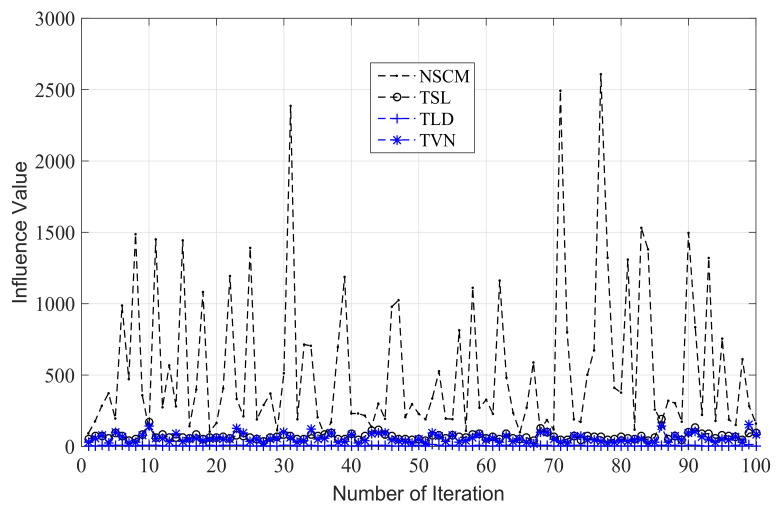
The influence value of arithmetic mean and total Bregman divergence median.

In order to analyze the robustness of total Bregman divergence median and arithmetic mean estimators, we inject a outlier with normalized Doppler frequency $f_{d}=0.1$ in 30 sample data, and compute the influence value according to (28), (29), (30), and (31). The results are shown in Figure 5. From Figure 5, we can know that the TLD median estimator has the smallest influence value, followed by the TSL median estimator. However, all of our proposed three estimators have smaller influence value than the arithmetic mean estimator, which is the NSCM estimator. These results imply that our proposed estimators are more robust than the NSCM estimator.

**Figure 6:**
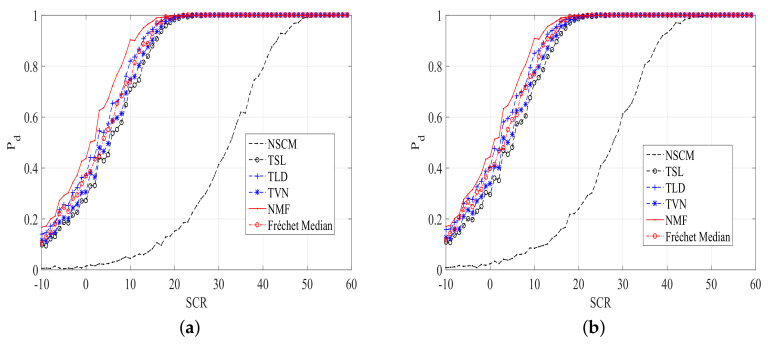
Pd versus SCR plots of ANMFs with the proposed estimators and the NSCM estimator, N=8,K=10. (**a**) $P_{fa}=10^{-5}$; (**b**) $P_{fa}=10^{-4}$.

**Figure 7:**
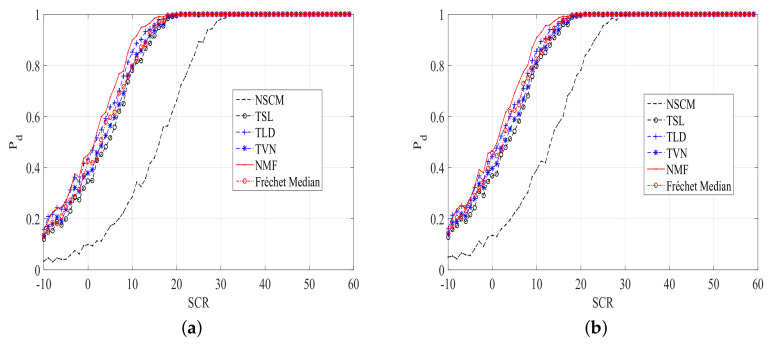
Pd versus SCR plots of ANMFs with the proposed estimators and the NSCM estimator, N=8,K=16. (**a**) $P_{fa}=10^{-5}$; (**b**) $P_{fa}=10^{-4}$.

To enhance the persuasiveness of the results, we inject a outlier with the $f_{dc}$ = 0.15, and SCR = 20 dB. Then, the plots of $P_{d}$ versus SCR of ANMF with different estimators are given in Figure 6 and Figure 7. It is clear from Figure 6 and Figure 7 that the proposed TSL, TLD, and TVN estimators outperform the NSCM in a contaminated clutter. Moreover, the performance of the Frechet median estimator is close to our proposed estimators.

## 6. Conclusions

In this work, we have proposed a covariance estimation method based on information geometry in a heterogeneous clutter. The problem of covariance estimation has been reformulated as the geometric median related to a measure. In particular, the three tBDs, including the TSL, the TLD, and the TVN, have been proposed on the Riemannian manifold. Then, we have derived the TSL, the TLD, and the TVN median estimators. At the analysis stage, the results of numerical experiments have highlighted that our proposed estimators outperform the NSCM estimator, and have similar performance to the Frechet median estimator in heterogeneous clutter.
